# Seroprevalence of porcine torovirus (PToV) in Spanish farms

**DOI:** 10.1186/1756-0500-5-675

**Published:** 2012-12-05

**Authors:** Julio Alonso-Padilla, Jaime Pignatelli, Meritxell Simon-Grifé, Susana Plazuelo, Jordi Casal, Dolores Rodríguez

**Affiliations:** 1 Department of Molecular and Cellular Biology, Centro Nacional de Biotecnología, CSIC, C/Darwin 3, Madrid, 28049, Spain; 2 Department of Molecular, Cellular and Developmental Neurobiology, Instituto Cajal, CSIC, Av. Doctor Arce 37, Madrid, 28002, Spain; 3 Centre de Recerca en Sanitat Animal (CReSA), UAB-IRTA and Department of Animal Health, Universitat Autonoma de Barcelona, Bellaterra, Barcelona, 08193, Spain; 4 Current address: Department of Microbiology, New York University School of Medicine, 341 East 25th ST, New York, 10010, USA

**Keywords:** Porcine torovirus, Seroprevalence, Pigs, Spain

## Abstract

**Background:**

Torovirus infections have been associated with gastroenteritis and diarrhea in horses, cows, pigs and humans, especially in young animals and in children. Although asymptomatic in a large percentage of cases, however toroviruses may pose a potential threat to worsen disease outcome in concurrent infections with other enteric pathogens. Previous studies based on the analysis of limited numbers of samples indicated high seroprevalences against porcine torovirus (PToV) in various European countries. The aim of this work was to perform a seroepidemiological survey of PToV in Spanish farms in order to define the seroprevalence against this virus.

**Results:**

Serum samples (n = 2664) from pigs of different ages were collected from 100 Spanish farms coming from 10 regions that concentrate 96.1% of the 3392 farms with 80 or more sows censused in Spain. Samples were screened by means of an indirect enzyme-linked immune-sorbent assay (ELISA) based on a recombinant PToV nucleocapsid protein as antigen. The analysis of the whole serum collection yielded a total of 95.7% (2550/2664) seropositive samples. The highest prevalence (99.6%, 1382/1388) and ELISA values (average O.D. ± standard deviation) were observed in the sows (1.03±0.36) and the lowest prevalence (59.4%, 98/165) and anti-PToV IgG levels (0.45±0.16) were found amongst 3-week-old piglets. Both ELISA reactivity values and seroprevalence percentages rose quickly with piglet’s age from 3 to 11 weeks of age; the seroprevalence was 99.3% (2254/2270) when only the samples from sows and pigs over 11-weeks of age were considered. Antibodies against PToV were detected in all analyzed farms.

**Conclusions:**

This report describes the results of the largest torovirus seroepidemiological survey in farmed swine performed so far. Overall, the seroprevalence against PToV in animals older than 11 weeks of age was >99%, indicating that this virus is endemic in pig herds from Spain.

## Background

Toroviruses (*Coronaviridae* family, *Nidovirales* order) are emergent viruses with a potential of zoonotic transmission, that can cause enteric disease and diarrhea in different animal species and in humans [1]. Torovirus genome is a large (~28 kb) single stranded RNA molecule of positive polarity. The non-structural proteins are encoded by the first two-thirds of the genome in two overlapping open reading frames (ORF1a and ORF1b), whilst the four structural proteins (spike, S; membrane, M; hemagglutinin-esterase, HE; and nucleocapsid, N) are encoded by the last third of the genome [1,2]. Four species have been described within the torovirus genus. The first to be recognized was the equine torovirus (EToV), also known as Berne virus (BEV) [3]. This is the prototype species of the genus as it is the only one adapted to grow in cell cultures. The bovine torovirus (BToV) was discovered a few years later [4], and its pathogenesis investigated by experimental infections of gnotobiotic calves [5,6]. That research made available a source of BToV, which facilitated the development of diagnostic tools to study its epidemiology [7]. The cell culture infectivity of some BToV isolates has been recently reported [8,9]. The presence of toroviral particles in human fecal samples and its association with enteric disease has been shown in several reports [10-12], but the molecular information available about the human torovirus (HToV) is still scarce. Porcine torovirus (PToV) particles were initially observed by electron microscopy in pig fecal samples [13], and its first molecular identification was made by Kroneman et al. [14]. In the same study, over 80% seroprevalence against PToV was found in adult sows in The Netherlands using a heterologous neutralization assay against EToV. Very high seroprevalences against PToV have also been observed on samples from Switzerland using a similar neutralization assay [15], and in Spain by means of neutralization as well as ELISA [16,17]. However, in the three above cited studies, the numbers of farms surveyed and of serum samples analyzed were low.

The objective of this study was to define the PToV seroprevalence in Spain through a designed seroepidemiological survey. Noteworthy, Spain is the second pig producer country in the European Union [18].

## Results

**Figure 1:**
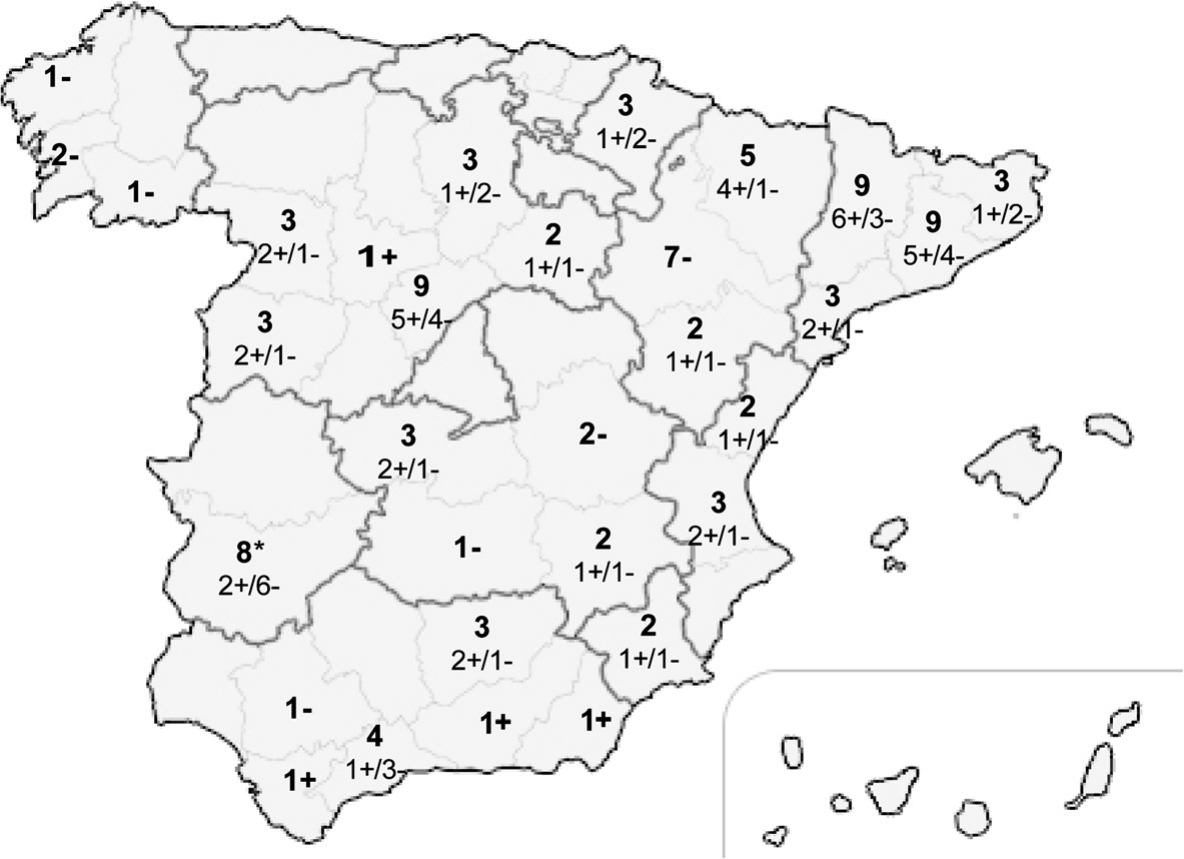
**Geographical distribution of the sampled pig farms (n = 100) throughout Spain.** The regions are delimited by dark grey lines and their provinces are delimited by light grey lines. The numbers in boldface indicate the total number of farms sampled at the indicated province, and the occurrence or absence of recent diarrheic outbreaks in the sampled farms is indicated by a “+” (n = 47) or “-” (n = 53) symbol, respectively. The asterisk symbol (*) indicates the only 8 sampled locations where pigs were raised outdoors.

A total of 2664 samples collected from 100 swine farms distributed over the entire territory of Spain (Figure 1) were tested on the basis of a previously described ELISA [16] for the presence of anti-PToV IgG using the nucleocapsid protein as antigen. All of them were intensive breeding farms except 8 locations, where pigs were raised in outdoor production facilities. Generally, 14 sows per farm were bled, and these serum samples formed the 52.1% (1388/2664) of the whole collection. Blood samples from pigs of distinct representative ages were also collected (Table 1), representing the sera from animals of 20 weeks of age the largest group amongst them (25.2%, 671/2664). Samples from pigs of 15 weeks (4.8%, 127/2664), 11 weeks (3.1%, 84/2664), 7 weeks (7.1%, 189/2664), 5 weeks (1.5%, 40/2664) and 3 weeks of age (6.2%, 165/2664) were also included in the study. From each farm, in addition to the samples from the 14 sows, at least 9 samples selected from 20-week-old pigs (in farrow-to-finish farms) or 7-week-old animals (in farrow-to-weaning farms) were also analyzed, while samples from younger animals were only analyzed in some of farms.

**Table 1:** Summary of the animal age groups included in the study and their seroprevalence against PToV

**Age**	**No.of sera**	**Positive**	**Prevalence** (**95%CI**)^**a**^	**O.D.**_**492**_ ± **s.d.**^**b**^
Sows	1388	1382	99.6% (99.0; 99.8)	1.03 ± 0.36
20 weeks	671	664	98.9% (97.8; 99.5)	0.85 ± 0.33
15 weeks	127	127	100.0% (96.5; 100.0)	1.02 ± 0.24
11 weeks	84	81	96.4% (89.6; 99.2)	0.73 ± 0.34
7 weeks	189	161	85.2% (79.4; 89.6)	0.52 ± 0.17
5 weeks	40	37	92.5% (79.4; 98.1)	0.59 ± 0.31
3 weeks	165	98	59.4% (51.8; 66.6)	0.45 ± 0.16
**Total**	2664	2550	95.7% (95.0; 96.5)	

^a^ CI, exact binomial confidence interval. ^b^ Mean O.D. _492_ values and their corresponding standard deviation.

The analysis of the whole serum collection yielded a total of 95.7% (2550/2664) seropositive samples. The reactivity values of the positive sera in the ELISA were positively correlated to the seroprevalence found in each age group analyzed (Table 1). A high prevalence and high O.D. values were observed in the sows. Both O.D. values and seroprevalence rose quickly with piglet’s age from 3 to 11 weeks. A prevalence of 59.4% (98/165) was found amongst 3-week-old piglets, and it reached an 85% in 7-week-old animals. Beyond 11-weeks of age seropositivity remained >99% (2254/2270). This antibody response along time is in accordance with previously reported studies [14,17].

**Figure 2:**
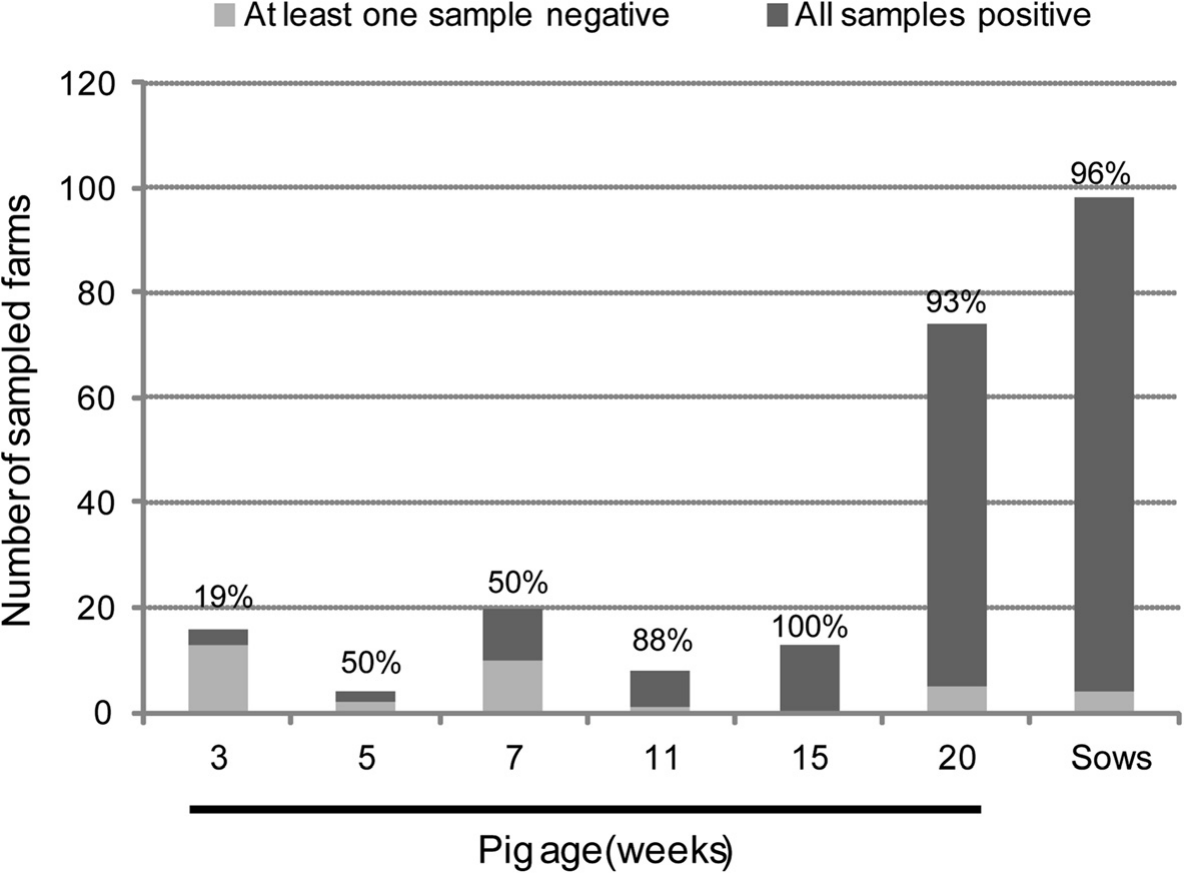
**Number of farms in which all animals sampled for a given age group were positive (dark grey) or at least one sample was seronegative (light grey).** The percentage of farms in which all samples from the indicated age group were positive is shown above each bar. Only those farms from which nine or more animals per age group were tested have been included in the graph.

All the farms surveyed had seropositive animals, and in 69 of them all the sera analyzed were positive. Figure 2 summarizes the numbers of farms in which all the animal sera tested from a given age were positive, versus the numbers of farms in which some negative samples were found. Only the farms from which 9 or more animals per age group were analyzed have been included in the figure. Depicted by ages, it was observed that all the samples from 11-, 15-, and 20-week-old pigs and sows were positive in a majority of the farms (Figure 2). In contrast, when only samples from 3- to 7-week-old piglets were considered, the percentages of farms with all the animals seropositive decreased, ranging from 19% (3-week-old piglets) to 50% (5-and 7-week-old piglets). These data indicated that the few negative sera found in the study were not concentrated in a particular set of PToV-free herds, and that the age-dependency of seropositivity was observed consistently on all the studied farms.

The farms included in the study could be classified in accordance to farm-level risk factors such as their recent history of diarrheic clinical signs (positive, n = 47; negative n = 53) or the breeding system used (farrow-to-weaning, n = 30; farrow-to-finish, n = 70). We did not find statistically significant differences on prevalence and average O.D. values on farms with recent history of diarrheic signs and those from farms without these signs; nor differences could be determined either related to the breeding system or when samples from animals raised outdoors (8 farms) were analyzed separately (data not shown). Overall, the prevalence and O.D. values obtained were very high and not statistically related to the farm clinical state, the breeding system employed, the region, and/or the biosecurity measures applied in the farms.

## Discussion

Based upon the availability of a cost-effective and feasible ELISA diagnostic tool for PToV, a large seroepidemiological survey was performed in Spanish swine herds. This ELISA methodology had been previously shown to be sensitive and specific for anti-PToV pig IgG antibodies, and showed a very good correlation with the EToV heterologous neutralization assay [16,17]. Sampling was designed to establish the seroprevalence in the Spanish farmed swine population. The samples included sera from a window of ages, from suckling piglets to sows of different parity number. However, since the main goal of this study was to determine the seroprevalence to PToV, the vast majority of samples belonged to adult animals, sows and 20-week-old pigs. In young animals, the levels of antibodies against PToV vary significantly with their age, as it has been previously described [14,17]. Newborn piglets receive maternal IgG antibodies through the colostrum, which levels decline soon and almost vanish around the time of weaning (3-weeks after birth). From then on, piglets develop their own immune response to the virus due to viral exposure, and IgG antibodies against PToV can be detected again by week 5 of age. Therefore, the analysis of adult pigs’ serology would reflect more faithfully the farm serological status.

After the analysis of 2664 field serum samples, almost all the animals of 11-weeks of age or over were found to have been exposed to the virus, independently of the breeding system used in the farms, the occurrence or not of recent diarrheic outbreaks, the region where farms were located or the biosecurity measures kept in them. The high seroprevalence results obtained here confirm previously reported observations from other studies where fewer samples were analyzed [14,16,17]. Although a low seropositivity to PToV has also been reported, that conclusion could have been due to the detection method used, which relied on a BToV antigen based ELISA [19].

Since this study includes serum samples from pigs of a wide window of ages, the obtained results provide an overview of the anti-PToV IgG response developed over time. The peak of anti-PToV IgG observed in the sows, the lower prevalence and antibody levels at weaning (3-weeks of age), and the seroprevalence and O.D. values increase observed thereafter (see Table 1) all fit in previously described anti-PToV IgG dynamics [14,17]. Accordingly, the differences observed regarding the response intensity in pigs under 11-week of age compared to older animals can be explained by the waning of the maternally-derived anti-PToV antibodies around weaning time, and the gradual development of piglets’ own immune response soon thereafter due to exposure to the virus once protection from maternal antibodies has vanished, as previously defined in detailed longitudinal seroepidemiological studies [14,17].

The overall serological pattern evidences a continuous spread of the virus in the farms, which could be due to recurrent infections of susceptible pigs [20], and those would also explain the immune response boosting effect observed in young pigs. This scenario would suggest a persistence of the infection on the farms, in which chronically infected adult animals could be acting as reservoirs as it has been hypothesized for other viral enteric infections [21]. Assessing PToV shedding by molecular detection in adult animals over time should be undertaken to analyze this hypothesis.

The impact of this high PToV seroprevalence in the pig production process still remains unknown. Although PToV infections are mostly subclinical, its pathogenesis and potential zoonotic implications are poorly understood [22]. It is important to bear in mind that the closely related BToV is considered a potential important threat for young and neonatal calves [7], on its own or in concurrent infections with other enteric viruses, such as rotavirus and astrovirus, that worsen the disease outcome [6]. In addition, BToV has been recently reported to affect adult dairy cattle production due to diarrhea outbreaks [23]. Moreover, the observed antigenic cross-reactivity between HToV and BToV could suggest the possibility of zoonotic transmission events [7]. Thus, in light of the apparent endemic character of PToV infection, pathogenesis studies with this virus, alone and in combination with other swine enteric viruses should be implemented.

## Conclusions

This is the largest seroepidemiological PToV survey performed so far. Taking together the high prevalence found and the antibody response observed in pigs from different ages, covering a window from suckling to adults, the results obtained undoubtedly confirm the broad dispersion of PToV in Spain and mark the endemic character of this infection in the pig population.

## Methods

### Survey instrument description

A cross-sectional survey was performed (2008–2009) to obtain serum samples representative from the Spanish pig population. A questionnaire was filled through an on-farm interview with the farmer to obtain general data (location, number of workers, presence of other domestic animal species), production data, facilities (including water supply and sewage system), biosecurity measures related to replacement stock, farm management and facilities and health status and vaccination schedule (including records of enteric disease outbreaks during the last year) [24].

Farm owners’ consent was obtained for the use of animals for this research. The ethical approval for the survey was provided by the Ethical and Animal Welfare Committee of the Universitat Autònoma de Barcelona and the Ethical and Animal Welfare Committee of the Government of Catalonia (December 13^th^, 2007; number of approval: 4226).

### Field sample collection

Sampling was restricted to Spanish regions where the pig census accounted for at least 2.5% of the total national census (the census was conducted by the Spanish Ministry of Agriculture in 1999/2000, and the last update before the survey was performed in 2007). With this restriction, 10 regions were included, accounting for 96.1% of the total Spanish pig farms that had a census of 80 sows or more (3392 farms). Considering a starting prevalence hypothesis of 50%, and setting the desired precision at ±10% and the confidence level at 95%, a sample size of 97 farms was obtained. For practical reasons, one hundred farms were considered, and the sampling was stratified by regions. In regions where the animal health authorities directly collaborated with the sampling (Cataluña, Navarra, Castilla-La Mancha, Extremadura, Galicia and Andalucía) the farms (58/100) were fully selected at random. In the other four regions (Aragón, Castilla y León, Murcia and Valencia), accounting for 42 farms, a convenience sampling based on the availability of swine practitioners was used to complete the selection of farms.

Animals were bled using a sterile collection system (Vacutainer®, Becton- Dickinson). Samples were transported to the laboratory under refrigeration (4°C) within 24–48 h of sampling. Blood samples were centrifuged at 400 x g for 15 min at 4°C and sera were stored at −80°C until further analysis.

### ELISA

The presence of specific IgG antibodies against PToV in the sera was tested using an indirect ELISA previously developed in our laboratory, which is based on the use of the highly conserved viral N protein produced as a recombinant antigen [16,17]. The ELISA had been optimized using PToV positive sera from naturally infected pigs and negative sera form caesarean-derived, colostrum-deprived pigs kept under germ free conditions, and the assay cut-off had been established after five independent experiments performed in different days at an optical density value at 492 nm (O.D. _492 nm_) < 0.270. In the absence of a gold standard assay for the detection of anti-PToV antibodies to establish the sensibility and specificity of the assay, the sample diagnostic performance of the ELISA was compared with that of the EToV heterologous neutralization assay used in previous reports [16,17]. Overall an 89.9% sample-level test agreement between the two assays was obtained. A single batch of recombinant N protein was used throughout the study and negative and positive control sera were included in each plate.

### Statistical analysis

Data entry and data coding were performed with Excel. ANOVA and χ^2^ tests were applied to evaluate the significance of association of each farm-level risk factor with each of the two outcomes: overall (across age groups) seroprevalence and the average O.D. value on the farm. Analyses were performed with SPSS 17.0. Confidence intervals for proportions were calculated with EpiCalc2000 (J.Gilman and M.Myatt, http://www.brixtonhealth.com/epicalc.html).

## Competing interests

The authors declare that they have no competing interests.

## Authors’ contributions

JAP participated in the design of the study, carried out some of the immunoassays, the analysis of the results and wrote the manuscript. JP participated in the design of the study, carried out some of the immunoassays and helped to draft the manuscript. MS participated in the selection of the farms and performed the sera collection. SPC performed most of the immunoassays. JC performed the selection of the farms, coordinated the sera collection and participated in the design of the study and the analysis of the results. DR conceived the study, participated in its design, coordination and draft of the manuscript. All authors read and approved the final manuscript.
